# The Effect of Spiritual Orientation and Perceived Stress on Heart Rate Variability and Electrocardiographic Parameters in Hypertensive Patients

**DOI:** 10.3390/healthcare14101316

**Published:** 2026-05-12

**Authors:** Funda Eldemir, İsa Ardahanlı

**Affiliations:** 1 Department of Elderly Care, Gülhane Training and Research Hospital, 06010 Ankara, Türkiye; funda.eldemir@sbu.edu.tr; 2 Department of Cardiology, Bilecik Şeyh Edebali University, 11230 Bilecik, Türkiye

**Keywords:** hypertension, psychophysiology, heart rate variability, perceived stress, spiritual orientation, autonomic regulation

## Abstract

**Background**: Hypertension is increasingly recognized as a complex psychophysiological condition in which psychological factors interact with autonomic regulation and cardiac electrical stability. This study aimed to investigate the associations of spiritual orientation, perceived stress, and self-efficacy with heart rate variability (HRV) and electrocardiographic (ECG) repolarization parameters in individuals with hypertension. **Methods**: A total of 200 participants were included, comprising 100 hypertensive patients and 100 age- and sex-matched healthy controls. HRV was assessed using time-domain indices (SDNN and RMSSD), while ECG parameters included heart rate, QRS duration, QT interval, Tp-e interval, and Tp-e/QT ratio. Psychosocial variables were evaluated using validated scales. Group comparisons, correlation analyses, and multivariate regression models were performed. **Results**: Compared with controls, hypertensive patients exhibited significantly lower SDNN (68.73 ± 10.74 vs. 82.85 ± 10.74 ms, *p* < 0.001, Cohen’s d = 1.52) and RMSSD (35.55 ± 8.36 vs. 44.17 ± 8.36 ms, *p* < 0.001, d = 1.18), along with higher heart rate (74.73 ± 9.12 vs. 68.72 ± 8.85 bpm, *p* < 0.001, d = 1.11) and increased repolarization parameters, including QT interval (407 ± 18.3 vs. 397.58 ± 17.9 ms, *p* < 0.001, d = −0.69), Tp-e interval (97.95 ± 10.2 vs. 90.94 ± 9.8 ms, *p* < 0.001, d = 0.89), and Tp-e/QT ratio (0.24 ± 0.02 vs. 0.23 ± 0.02, *p* < 0.001). Spiritual orientation was positively correlated with SDNN (r = 0.274, *p* < 0.001) and RMSSD (r = 0.242, *p* < 0.001) and negatively correlated with heart rate (r = −0.277, *p* < 0.001), Tp-e (r = −0.256, *p* < 0.001), and Tp-e/QT ratio (r = −0.258, *p* < 0.001). Perceived stress showed inverse correlations with HRV indices and positive associations with repolarization parameters. In multivariate analysis, spiritual orientation remained an independent predictor of higher HRV indices, whereas perceived stress independently predicted a longer Tp-e interval and lower HRV. **Conclusions**: Spiritual orientation and stress-related factors are significantly associated with both autonomic function and cardiac repolarization in hypertension. These findings support a psychophysiological model in which psychosocial resources and stress responses jointly influence cardiovascular regulation. Integrating psychosocial assessment into hypertension management may provide additional insights beyond traditional risk factors.

## 1. Introduction

Hypertension (HT) remains one of the leading causes of cardiovascular morbidity and mortality worldwide [1,2]. Hypertension represents a major public health concern in Turkey, with prevalence rates reported to be comparable to or higher than global averages. National studies have highlighted the growing burden of hypertension and its associated cardiovascular complications, emphasizing the need for a more comprehensive understanding of both biomedical and psychosocial determinants. In this context, investigating the interplay between psychological factors and objective cardiovascular markers may provide valuable insights for improving patient management in the Turkish population. Although HT is primarily defined in clinical practice by blood pressure levels, current data indicate that this condition is not merely a hemodynamic disorder; it is a complex cardiovascular syndrome characterized by autonomic nervous system dysfunction, neurohumoral activation, endothelial dysfunction, and inflammatory processes [3,4]. In particular, chronic increase in sympathetic nervous system activity and decrease in parasympathetic (vagal) tone play a central role in the development and maintenance of hypertension [5].

Heart rate variability (HRV) is widely used in clinical and research settings as a non-invasive, quantitative indicator of autonomic nervous system activity [6]. Decreased HRV indicates a disruption of sympathovagal balance and a reduced adaptive capacity of the cardiovascular system to physiological stressors [6,7]. Low HRV in hypertensive individuals; Increased cardiovascular risk, target organ damage, and poor prognosis have been associated with it [7,8]. Therefore, HRV is considered not only an accompanying finding in hypertension but also an important marker of the disease’s pathophysiological depth. Autonomic instability in hypertension extends beyond heart rate variability and also affects cardiac electrical stability [9]. Ventricular repolarization heterogeneity is an important component of the arrhythmogenic substrate and can be indirectly assessed by electrocardiographic (ECG) parameters. Parameters such as QT duration, Tp-e interval, and Tp-e/QT ratio have received increasing attention in recent years as practical indicators reflecting transmural repolarization dispersion [10].

Prolongation in these parameters is associated with increased sympathetic activity, baroreflex impairment, and myocardial remodeling, and may predispose to an increased risk of arrhythmias in hypertensive individuals [9,10]. Psychosocial stress is a significant environmental factor known to influence the development and control of hypertension [11]. Increased perceived stress alters autonomic balance towards sympathetic dominance by increasing cortisol and catecholamine levels via the hypothalamo-pituitary–adrenal axis and the sympathoadrenal system [11,12]. This process can result in decreased heart rate variability, increased heart rate, and impaired cardiac repolarization dynamics. Indeed, the adverse effects of stress on heart rate and its relationship with QT dynamics have been demonstrated in various clinical and experimental studies [6,10]. However, the cardiovascular effects of stress depend not only on the presence of the stressor but also on the individual’s capacity to cope with stress and their psychosocial resources. In this context, self-efficacy perception is an important psychosocial determinant reflecting an individual’s ability to cope with stressful situations and maintain health-related behaviors [12]. Low self-efficacy perception can negatively affect treatment adherence and lifestyle changes in chronic diseases, indirectly impairing both blood pressure control and autonomic balance [11,12]. In recent years, the concepts of spiritual orientation and spirituality have received increasing attention among psychosocial factors associated with cardiovascular health [13]. Spiritual orientation is closely related to an individual’s ability to attribute meaning to their life, feelings of hope and acceptance, perceptions of social support, and stress-coping strategies. An increasing number of studies suggest that spiritual or mental well-being can reduce perceived stress, increase psychological resilience, and, in some cases, improve cardiovascular outcomes [13,14]. Beyond autonomic regulation, psychosocial factors may also influence cardiac electrical stability through shared neurobiological pathways. Chronic stress and reduced psychological resilience can activate the hypothalamic–pituitary–adrenal axis and increase sympathetic tone, which not only suppresses vagal activity, as reflected in HRV, but also alters ventricular repolarization dynamics. Increased sympathetic activity and impaired baroreflex sensitivity have been associated with prolonged QT interval and increased Tp-e duration, reflecting greater repolarization heterogeneity. Conversely, spiritual orientation may act as a buffering factor by enhancing emotional regulation, reducing stress reactivity, and promoting parasympathetic dominance. Therefore, spirituality and stress-related constructs may simultaneously affect both HRV and ECG-based repolarization parameters through integrated psychophysiological mechanisms. However, the effects of spiritual orientation on autonomic nervous system function and cardiac electrical stability have been evaluated in only a limited number of studies, mostly using indirect measures. In the current literature, studies that comprehensively address psychosocial factors such as spiritual orientation, perceived stress, and self-efficacy in hypertensive individuals, along with heart rate variability and ventricular repolarization indicators, are limited [14,15]. In particular, elucidating the potential effects of spiritual orientation on objective cardiac physiological parameters can contribute to a better understanding of the biopsychosocial nature of hypertension.

This study aims to evaluate the relationship between spiritual orientation, perceived stress, and low self-efficacy levels with heart rate variability and electrocardiographic repolarization parameters in individuals diagnosed with hypertension, and to reveal the possible pathophysiological basis of these relationships.

## 2. Methods

### 2.1. Study Design and Population Characteristics

This study is a cross-sectional, observational study designed to evaluate the effects of spiritual orientation and perceived stress on heart rate variability (HRV) and ECG parameters in individuals diagnosed with hypertension. The study aims to examine the multidimensional relationships among psychosocial factors, cardiovascular autonomic regulation, and cardiac electrical stability. A total of 200 individuals were included in the study. 100 of the participants were patients diagnosed with HT, and 100 were healthy control group individuals matched for age and gender without a known history of HT or cardiovascular disease. The diagnosis of HT was confirmed based on previously established clinical diagnosis and/or regular use of antihypertensive treatment. Individuals in the control group did not have a history of hypertension, coronary artery disease, heart failure, clinically significant arrhythmias, or structural heart disease. Exclusion criteria for both groups were: known coronary artery disease, heart failure, significant valvular disease, persistent or paroxysmal arrhythmias, structural heart disease, chronic inflammatory or systemic diseases, history of malignancy, and use of drugs known to have a significant effect on the autonomic nervous system. Patients receiving antihypertensive treatment were not excluded from the study. 55.5% of the participants were female (*n* = 111) and 44.5% were male (*n* = 89). The mean age of the participants was 50.0 ± 8.9 years, and the mean BMI was 28.4 ± 3.4 kg/m^2^. BMI classification was based on World Health Organization criteria [16]. Participants were recruited using a consecutive sampling approach from the outpatient cardiology clinic of a tertiary care hospital. All eligible patients presenting during the study period were screened for inclusion according to predefined criteria. The recruitment was conducted over a defined period, and both hypertensive patients and control participants were enrolled in parallel. The control group consisted of individuals without known cardiovascular disease who were matched for age and sex. The recruitment process was designed to minimize selection bias; however, no formal record of refusal rate was maintained. Therefore, the possibility of selection bias cannot be entirely excluded and is acknowledged as a limitation of the study.

### 2.2. Electrocardiographic Evaluation

**Figure 1:**
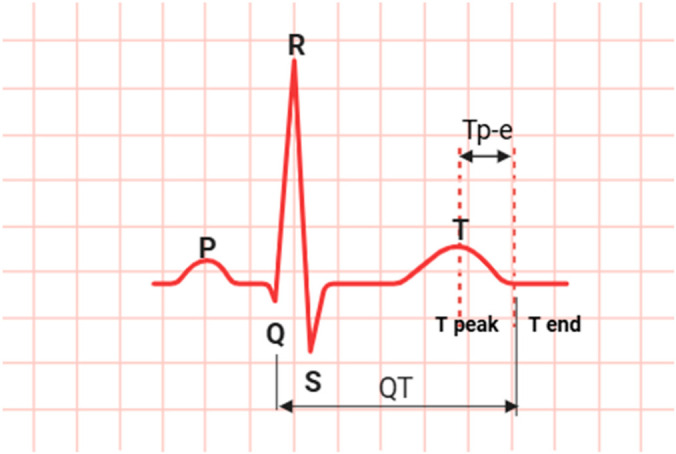
Schematic ECG tracing showing the QT interval and the Tp-e interval. The QT interval extends from the onset of the QRS complex to the end of the T wave, while the Tp-e interval is measured from the T-wave peak to its end and reflects ventricular repolarization dispersion.

All participants underwent 12-lead surface ECG under standard conditions in a quiet, controlled environment after at least 5 min of rest. ECG recordings were digitized and analyzed at 300× magnification. Heart rate (beats/min), QRS duration (ms), QT interval (ms), Tp-e interval (ms), and Tp-e/QT ratio were measured from the ECG recordings. The QT interval was measured by averaging at least 3 consecutive complexes and corrected for heart rate. The Tp-e interval was defined as the time between the peak and end of the T wave and was considered an indicator of cardiac repolarization heterogeneity. The Tp-e/QT ratio was included in the analyses as a heart rate-independent indicator of repolarization (Figure 1).

Heart rate variability analysis was performed using time-domain parameters from resting ECG recordings. SDNN (standard deviation normal–normal range), reflecting global autonomic activity, and RMSSD (root mean square of successive differences), an indicator of parasympathetic activity, were calculated. HRV parameters were evaluated as quantitative indicators of cardiac autonomic regulation.

### 2.3. Psychosocial Measurements

Participants’ spiritual orientation levels were assessed using a spiritual orientation scale for which Turkish validity and reliability studies have been previously conducted [17]. The Spiritual Orientation Scale used in this study has been validated in the Turkish population, demonstrating good internal consistency (Cronbach’s alpha ≈ 0.85–0.90). The scale includes items reflecting meaning in life, connectedness, and inner peace (e.g., “I feel a sense of purpose in my life”). Perceived stress was assessed using the Perceived Stress Scale (PSS), which has been validated in Turkish populations with acceptable reliability (Cronbach’s alpha ≈ 0.80). The scale includes items such as “In the last month, how often have you felt unable to control important things in your life?” Self-efficacy was evaluated using a validated self-efficacy scale adapted for Turkish populations, with reported Cronbach’s alpha values typically above 0.80. Example items assess individuals’ confidence in coping with challenging situations. High scores on the scale indicate a strong spiritual orientation. Perceived stress level was assessed using a standard perceived stress scale that measures stress-related discomfort; higher scores indicated greater perceived stress. In addition, the perception of insufficient self-efficacy, which indirectly reflects individuals’ capacity to cope with stress, was measured through a validated scale.

### 2.4. Statistical Analysis

Statistical analyses were performed using JASP (version 0.19.0) and IBM SPSS Statistics software. Continuous variables were presented as mean ± standard deviation or median (interquartile range), while categorical variables were presented as number and percentage. Normality of the data distribution was assessed using the Shapiro–Wilk test, as well as skewness and kurtosis values. Variables showing normal distribution were analyzed using parametric tests, while non-normally distributed variables were analyzed using non-parametric methods. Specifically, independent-samples t-tests were used for normally distributed variables, and Mann–Whitney U tests were applied for non-normally distributed variables.

Correlation analyses were performed using Pearson’s correlation coefficient for normally distributed variables and Spearman’s rank correlation coefficient for non-normally distributed variables. Given the multiple comparisons performed in the study, the results were interpreted cautiously to reduce the risk of Type I error. Although formal correction methods (e.g., Bonferroni adjustment) were not applied due to the exploratory nature of the study, emphasis was placed on effect sizes and consistency of findings across related variables. To further strengthen the analytical framework, multivariate linear regression analyses were conducted to identify independent predictors of HRV and ECG parameters after adjusting for relevant covariates. Parametric tests were preferred for variables that met the standard distribution assumption, and nonparametric tests were preferred for variables that did not. The relationships among ECG parameters, HRV measures, spiritual orientation, perceived stress, and insufficient self-efficacy scores were examined using Pearson or Spearman correlation analyses, depending on the data distribution. Independent-samples t-test or Mann–Whitney U test was used for comparisons between two groups based on hypertension status; One-Way ANOVA or Kruskal–Wallis test was applied for comparisons based on age and BMI subgroups. In parametric analyses, the effect size was calculated as Cohen’s d; in nonparametric analyses, the biserial correlation coefficient was used. A statistical significance level of *p* < 0.05 was accepted for all analyses. Effect sizes were interpreted according to Cohen’s criteria, where d = 0.2 represents a small effect, d = 0.5 a medium effect, and d = 0.8 a large effect. A priori sample size calculation was not performed due to the observational and exploratory design of the study. However, a post hoc power analysis was conducted based on the observed differences in primary HRV parameters (SDNN and RMSSD) between groups. The analysis indicated that the study had >90% statistical power to detect medium-to-large effect sizes at an alpha level of 0.05. Therefore, the sample size of 200 participants was considered adequate for the primary analyses performed in this study. Potential confounding variables, including smoking status, caffeine intake, physical activity level, and comorbid conditions such as diabetes and psychological disorders, were recorded during participant assessment. Although these variables were not used as primary grouping factors, they were considered in the analytical framework and included as covariates in multivariate regression models where appropriate.

Heart rate variability (HRV) analysis in this study was based on time-domain parameters derived from resting ECG recordings. While frequency-domain and nonlinear HRV analyses provide additional insights into autonomic regulation, time-domain measures such as SDNN and RMSSD are widely accepted and clinically validated indicators of global and parasympathetic autonomic activity, particularly in observational clinical studies.

### 2.5. Ethical Approval

This study was approved by the Non-Interventional Clinical Research Ethics Committee of Bilecik Şeyh Edebali University Faculty of Medicine (approval date: 26 February 2025; decision number: 2025/2–10), in accordance with the principles of the Declaration of Helsinki and relevant national regulations. All participants were informed about the purpose and procedures of the study, and written informed consent was obtained prior to participation.

## 3. Results

### 3.1. Findings from an Examination of the Relationship Between ECG Measurements, Spirituality Score, Stress Distress Score, and Insufficient Self-Efficacy Score

Table 1 shows that the SDNN value has a high level of significant positive correlation with the RMSSD value; a low level of significant positive correlation with the Spirituality Score; and a low level of significant negative correlation with HR, Stress Discomfort Score, and Insufficient Self-Efficacy Score. The RMSSD value shows a negative, low-level significant correlation with HR, Tp-e, Tp-e/QT, Stress Discomfort Score, and Insufficient Self-Efficacy Score, and a positive, low-level significant correlation with the Spirituality Score. The HR value has a positive and high level of significant correlation with other ECG values such as QRS duration, QT interval, Tp-e interval, and Tp-e/QT ratio; a positive and low level of significant correlation with the Stress Discomfort Score; and a negative and low level of significant correlation with the Spirituality Score. The QRS value shows a moderate, positive level of significant correlation with QT, Tp-e, and Tp-e/QT, and a low, negative level of significant correlation with the Spirituality Score. While there is a positive and highly significant correlation between QT and Tp-e and between Tp-e/QT values, there is a positive, low-level significant correlation with the Stress Discomfort Score and a negative, low-level significant correlation with the Spirituality Score. A positive and highly significant correlation was observed between Tp-e and Tp-e/QT; a negative, low-level significant correlation with the Spirituality Score; and a negative, low-level significant correlation with the Stress Discomfort Score. A negative, low-level, significant correlation was found between Tp-e/QT and the Spirituality Score, and a positive, low-level, significant correlation with the Insufficient Self-Efficacy Score. A negative and moderately significant correlation was found between the Spirituality Score and stress scores. It was concluded that stress scores showed a positive, moderately significant correlation.

### 3.2. Findings Regarding Demographic Variables

Table 2 shows a statistically significant difference (*p* < 0.05) between ECG parameters based on the patient status variable. For SDNN and RMSSD, the mean scores of control participants were higher than those of hypertensive patients (SDNN: 82.85 vs. 68.73 ms, *p* < 0.001; RMSSD: 44.17 vs. 35.55 ms, *p* < 0.001). Conversely, HR, QRS, QT, Tp-e, and Tp-e/QT values were significantly higher in hypertensive patients compared with controls. When examining the effect sizes, it was observed that QT and Tp-e/QT values had moderate effect sizes, while other ECG parameters had large effect sizes.

A statistically significant difference (*p* < 0.05) was observed between spirituality scores based on the patient status variable. The mean score of non-patient participants was significantly higher than that of patient participants. The effect size for this analysis was large. Stress scores differed significantly (*p* < 0.05) by patient status, with both stress scores higher in patient participants than in non-patient participants.

When Table 3 is examined, no significant differences were found in any of the scores (SDNN, RMSSD, HR, QRS, QT, Tp-e, Tp-e/QT, Spirituality, Stress Discomfort, Insufficient Self-Efficacy) across gender (*p* > 0.05).

When Table 4 is examined, no significant differences were found between age groups for all variables (SDNN, RMSSD, HR, QRS, QT, Tp-e, Tp-e/QT, Spirituality, Stress Discomfort, Insufficient Self-Efficacy) (*p* > 0.05).

Multivariate linear regression analyses were performed to determine independent predictors of HRV and ECG parameters after adjustment for age, sex, BMI, and hypertension status (Table 5). Spiritual orientation remained an independent positive predictor of SDNN (β = 0.23, *p* < 0.001) and RMSSD (β = 0.21, *p* < 0.001). In contrast, perceived stress independently predicted lower HRV indices and higher repolarization parameters, including Tp-e interval (β = 0.17, *p* = 0.004) and Tp-e/QT ratio (β = 0.16, *p* = 0.006). Insufficient self-efficacy showed weaker but borderline significant associations with selected parameters. Hypertension status remained a strong independent predictor across all models. These findings indicate that psychosocial factors are independently associated with both autonomic regulation and ventricular repolarization beyond traditional clinical variables.

## 4. Discussion

In this cross-sectional study, the relationship between spiritual orientation, perceived stress, and insufficient self-efficacy levels with heart rate variability (HRV) and electrocardiographic repolarization indicators was evaluated in individuals diagnosed with hypertension. The findings suggest that HRV is significantly reduced in the presence of hypertension, parameters such as QT, Tp-e, and Tp-e/QT, which reflect repolarization heterogeneity, are increased, spiritual orientation is associated with higher HRV and a more favorable repolarization profile, while perceived stress and insufficient self-efficacy are correlated with autonomic imbalance and electrical instability indicators. These findings indicate that hypertension is not only a hemodynamic disorder but also a complex syndrome intertwined with autonomic dysfunction, baroreflex impairment, neurohumoral activation, and inflammatory processes [3,18].

In HT, autonomic nervous system dysfunction and sympathetic overactivation are key mechanisms in both maintaining blood pressure and causing target organ damage. Increased sympathetic nervous activity creates a vicious cycle that fuels hypertension through peripheral vasoconstriction, renal sodium retention, activation of the renin–angiotensin–aldosterone system (RAAS), and cardiac remodeling. In recent years, the association of sympathetic overactivity with endothelial dysfunction and vascular damage has been more strongly emphasized; these processes increase arterial stiffness, reduce baroreceptor sensitivity, and further disrupt autonomic balance [19]. Therefore, decreased HRV in hypertension can be considered not merely an “accompanying finding” but an autonomic phenotype at the center of the pathophysiology.

Time-domain measures of heart rate variability (HRV), specifically SDNN and RMSSD, are commonly utilized in clinical research as indicators of global autonomic modulation and, more specifically, parasympathetic (vagal) activity. Decreased SDNN and RMSSD in hypertension suggest a shift in sympathovagal balance towards sympathetic activity, weakening of the vagal brake, and decreased physiological resilience of the cardiovascular system to environmental/psychological stressors. Compared with previous studies, the magnitude of HRV reduction observed in our hypertensive cohort appears to be clinically meaningful. For instance, prior research has reported moderate reductions in SDNN and RMSSD among hypertensive individuals; however, the effect sizes observed in our study were generally moderate to large, suggesting a more pronounced autonomic imbalance in our population. These differences may be attributable to variations in population characteristics, comorbidity burden, or measurement protocols.

Similarly, the observed increases in repolarization parameters, such as Tp-e and Tp-e/QT, are consistent with the existing literature; however, the strength of the associations in our study suggests a potentially stronger link between autonomic dysfunction and electrical instability. While some studies report weaker correlations between HRV and repolarization indices, our findings indicate a more integrated relationship between these domains. Importantly, discrepancies across studies may reflect differences in methodology, including short-term versus long-term HRV assessment, heterogeneity in antihypertensive treatment, and variability in psychosocial measurements. These factors should be considered when interpreting and comparing results across populations. In this context, current studies demonstrating hypertension-related autonomic impairment through HRV show that HRV can also be accompanied by chronic inflammation and disease burden [20,21]. Another noteworthy aspect of the study is the higher repolarization markers, such as QT, Tp-e, and Tp-e/QT, in the hypertension group. Ventricular repolarization heterogeneity is a significant component of the arrhythmogenic substrate, and the Tp-e interval and the Tp-e/QT ratio are suggested as practical ECG indicators reflecting transmural repolarization dispersion. Because the Tp-e/QT ratio is less variable than heart rate, it is considered a valuable index for capturing repolarization imbalance. The growing number of studies investigating the relationship between Tp-e and Tp-e/QT and prognosis across different clinical settings supports the potential of these indicators to reflect “electrical fragility” [22,23,24].

The negative correlation of perceived stress with HRV and its positive correlation with repolarization parameters strengthen the importance of the psychobiological stress response in hypertension. Acute and chronic stress affect the cardiovascular system through mechanisms such as increased cortisol via the hypothalamo-pituitary–adrenal (HPA) axis, increased catecholamines via the sympathoadrenal system, enhanced inflammation, and impaired endothelial function [11]. A broad literature supports the effect of stress on reducing HRV; meta-analytic data show that psychological stress may be associated with decreases in HRV parameters (especially in the RMSSD/SDNN axis) [25].

The role of stress in repolarization parameters is explained by the hypothesis that it can trigger changes, such as QT/QTc prolongation, with a sudden shift in autonomic tone toward sympathetic dominance. Studies evaluating the effects of acute mental stress on QT dynamics have shown that different cardiac electrical responses may occur depending on the type and severity of the stress. It has been reported that QTc prolongation and changes in T wave morphology can be observed, especially with increased sympathetic activity [26]. In this context, the correlation between stress and repolarization indicators in our study establishes a biologically consistent bridge between sympathetic dominance and repolarization heterogeneity in hypertension.

The increase in perceived inadequate self-efficacy, the decrease in HRV, and its positive relationship with stress are also clinically significant. Self-efficacy is a psychosocial determinant that affects an individual’s capacity to cope with stressors, maintain healthy behaviors, and adhere to treatment. In a chronic disease such as hypertension, low self-efficacy can negatively affect behavioral axes such as lifestyle adjustment, sleep patterns, physical activity, and medication adherence, indirectly disrupting both blood pressure control and autonomic balance [27]. Therefore, self-efficacy can be considered not only a psychological outcome but also a marker that touches the behavioral and autonomic layers of the cardiovascular risk architecture. The positive relationship between spiritual orientation and SDNN/RMSSD and the negative relationship in the QT–Tp-e axis are among the most original findings of the study. Spirituality can increase psychological resilience by fostering meaning, hope, acceptance, social bonding, and stress-coping strategies. On the psychophysiological level, it is suggested that spiritual well-being can suppress the HPA axis activation by reducing stress reactivity, supporting vagal tone, and limiting sympathetic overactivity [14]. This approach is consistent with current AHA-focused assessments discussing the relationship between religious/spiritual orientation and cardiovascular health [28].

Current studies show that spiritual or mind–body-based practices can have measurable effects on autonomic function. For example, heart-focused meditation/breathing-based interventions have been reported to increase vagal activity and improve HRV measures [29]. Furthermore, the increasing number of randomized studies reporting that “spirituality-based” interventions in hypertension can have positive effects on blood pressure and endothelial function supports the idea that spiritual orientation may be a physiological axis associated not only with subjective well-being but also with vascular function and autonomic balance [30].

Regarding repolarization parameters, the growing evidence that Tp-e and the Tp-e/QT ratio may be associated with increased arrhythmia risk and poor prognosis across different clinical scenarios underscores their potential importance in hypertension [23,24]. These indicators may be a standard electrical output of processes such as sympathetic overactivity, baroreflex impairment, microvascular dysfunction, and hypertension-related myocardial remodeling. Baroreflex dysfunction is particularly noteworthy as a mechanism that fuels repolarization heterogeneity by increasing both autonomic balance and hemodynamic fluctuations in hypertension [31]. The absence of significant differences in subgroup analyses according to gender, age, and BMI suggests that the observed relationship pattern may be independent of these demographic variables in our sample. An important consideration in interpreting the present findings is the potential influence of antihypertensive medications on autonomic and electrophysiological parameters. Beta-blockers, for instance, are known to increase vagal activity and reduce heart rate, potentially enhancing HRV indices, while certain calcium channel blockers and other agents may also influence ventricular repolarization dynamics. Therefore, part of the observed associations may be partially modulated by pharmacological effects.

Importantly, the multivariate regression analyses demonstrated that spiritual orientation and perceived stress remained independently associated with both HRV and repolarization parameters after adjustment for major clinical variables, suggesting that these relationships are not solely attributable to traditional confounders. However, residual confounding related to medication type and dosage cannot be completely excluded and should be addressed in future studies [32]. Therefore, future studies must address the impact of potential confounders in more detail, either through sensitivity analyses by drug class or through multivariate models.

In conclusion, this study’s findings demonstrate that stress-coping resources (such as spiritual orientation and self-efficacy) in hypertensive individuals may be significantly associated with cardiac autonomic function and repolarization stability. In addition to biomedical risk factors, psychosocial assessment and stress-reducing interventions can provide a complementary strategy in hypertension management, particularly in patients with significant autonomic dysfunction. These findings suggest that psychosocial assessment may complement traditional cardiovascular risk evaluation and contribute to a more comprehensive management approach in hypertensive patients.

## 5. Limitations

The cross-sectional design of the study does not allow for establishing a causal relationship. Since psychosocial measures rely on self-report scales, there is a risk of response bias. HRV and ECG measurements are single-time; 24 h Holter HRV or ambulatory blood pressure monitoring was not used. The potential effects of antihypertensive treatment classes and doses on HRV/repolarization were not modeled in detail. Therefore, confirmation of the findings is needed with larger sample sizes, prospective studies, and, if possible, intervention designs. Additionally, electrocardiographic measurements were obtained under standardized conditions; however, limitations due to equipment precision and measurement variability cannot be entirely ruled out.

Future studies should include larger and more diverse populations, incorporate long-term HRV monitoring, and examine the role of pharmacological treatment in greater detail. In addition, further research is needed to explore how psychosocial interventions may improve cardiovascular outcomes in hypertensive patients.

## 6. Conclusions

This study revealed that spiritual orientation and perceived stress levels showed significant correlations with heart rate variability and electrocardiographic repolarization indicators in individuals diagnosed with hypertension. The association of spiritual orientation with higher parasympathetic activity and a more stable cardiac repolarization profile, and conversely, the co-occurrence of perceived stress and inadequate self-efficacy with autonomic instability and electrical instability indicators, supports the biopsychosocial nature of hypertension. These findings suggest that considering psychosocial and spiritual dimensions in hypertension management can contribute to cardiovascular risk assessment.

Autonomic nervous system dysfunction and cardiac electrical instability in hypertensive individuals are multidimensional processes that cannot be explained solely by blood pressure levels. The findings of this study indicate that perceived stress load and stress-coping resources (such as spiritual orientation and self-efficacy) may be associated with heart rate variability and repolarization parameters. In clinical practice, considering psychosocial stress levels and coping mechanisms in the evaluation of hypertensive patients suggests that, in addition to pharmacological treatments, stress-reduction, mindfulness, or spiritually based approaches can play a complementary role. These findings should be interpreted in light of the study limitations, and further prospective studies with comprehensive autonomic assessment and detailed control of confounding variables are needed to confirm these associations.

## Figures and Tables

**Table 1:** Pearson and Spearman–Brown Correlation Test Results for Examining the Relationship Between ECG Values, Spirituality Score, Stress Discomfort Score, and Insufficient Self-Efficacy Score.

Variable	DNN (ms)	RMSSD (ms)	HR (bpm)	QRS (ms)	QT (ms)	Tp-e (ms)	Tp-e/QT	SS	SDS	ISES
SDNN (ms)	–									
RMSSD (ms)	0.866 ***	–								
HR (bpm)	−0.215 **	−0.275 ***	–							
QRS (ms)	−0.100	−0.053	0.561 ***	–						
QT (ms)	−0.092	−0.108	0.872 ***	0.501 ***	–					
Tp-e (ms)	−0.160 *	−0.238 ***	0.903 ***	0.555 ***	0.930 ***	–				
Tp-e/QT	−0.148 *	−0.166 *	0.862 ***	0.543 ***	0.826 ***	0.962 ***	–			
SS	0.274 ***	0.242 ***	−0.277 ***	−0.229 ***	−0.173 *	−0.256 ***	−0.258 ***	–		
SDS	−0.235 ***	−0.227 ***	0.184 **	0.132	0.149 *	0.156 *	0.134	−0.316 ***	–	
ISES	−0.176 *	−0.157 *	0.122	0.062	0.125	0.156	0.140 *	−0.309 ***	0.527 ***	–

Abbreviations: HR: Heart rate, SS: Spirituality Score, SDS: Stress Distress Score, ISES: Insufficient Self-Efficacy Score, * *p* < 0.05, ** *p* < 0.01, *** *p* < 0.001.

**Table 2:** Comparison of ECG Parameters, Spirituality, Perceived Stress, and Self-Efficacy Levels in Patient and Control Groups.

Variables	Groups	Mean	t	SD	U	z	*p*	Cohen d	Point-Biserial Correlation
SDNN (ms)	Control	82.85	10.74	198			<0.001	1.519	
	HT	68.73							
RMSSD (ms)	Control	44.17	8.36	198			<0.001	1.183	
	HT	35.55							
HR (bpm)	Control	68.72	−7.84	198			<0.001	−1.109	
	HT	74.73							
QRS (ms)	Control	94.62	−9.39	198			<0.001	−1.328	
	HT	100.46							
QT (ms)	Control	397.58	−4.87	198			<0.001	−0.689	
	HT	407							
Tp-e (ms)	Control	90.94	−6.31	198			<0.001	−0.892	
	HT	97.95							
Tp-e/QT	Control	0.23			2717	−5.601	<0.001		−0.457
	HT	0.24							
SS	Control	85.51			7769	−6.768	<0.001		0.554
	HT	71.59							
SDS	Control	16.10			3057.5	−4.762	<0.001		−0.388
	HT	18.64							
ISES	Control	11.19			3663	−3.286	0.001		−0.267
	HT	12.33							

Abbreviations: HR: Heart rate, SS: Spirituality Score, SDS: Stress Distress Score, ISES: Insufficient Self-Efficacy Score.

**Table 3:** Comparison of ECG Parameters and Spirituality, Perceived Stress, and Self-Efficacy Levels by Gender.

Variables	Gender	*n*	Mean	t	SD	U	Z	*p*
SDNN (ms)	Male	89	75.96	0.18	198			0.85
	Female	111	75.65					
RMSSD (ms)	Male	89	40.05	0.28	198			0.77
	Female	111	39.71					
HR (bpm)	Male	89	71.22	−1.02	198			0.31
	Female	111	72.12					
QRS (ms)	Male	89	97.41	−0.29	198			0.76
	Female	111	97.64					
QT (ms)	Male	89	401.41	−0.72	198			0.46
	Female	111	402.95					
Tp-e (ms)	Male	89	93.88	−0.82	198			0.41
	Female	111	94.89					
Tp-e/QT	Male	89	0.23			4632.5	−0.76	0.45
	Female	111	0.23					
SS	Male	89	78.13			4757.5	−0.45	0.65
	Female	111	78.88					
SDS	Male	89	16.98			4595	−0.85	0.39
	Female	111	17.67					
ISES	Male	89	11.596			4773.5	−0.41	0.68
	Female	111	11.892					

Abbreviations: HR: Heart rate, SS: Spirituality Score, SDS: Stress Distress Score, ISES: Insufficient Self-Efficacy Score.

**Table 4:** Comparison of ECG Parameters, Spirituality, Perceived Stress, and Self-Efficacy Levels by Age Groups (One-Way ANOVA and Kruskal–Wallis H Test).

Variables	Age (years)	*N*	Mean	Sum of Squares	F	SD	***X*** ^2^	*P*
SDNN (ms)	36–46	73	34.55	69.11	0.25	2		0.77
	47–57	76	137.15	27,018.64		197		
	58–68	51		27,087.75		199		
RMSSD (ms)	36–46	73	62.18	124.37	0.87	2		0.42
	47–57	76	71.51	14,088.28		197		
	58–68	51		14,212.66		199		
HR (bpm)	36–46	73	15.72	31.45	0.41	2		0.66
	47–57	76	38.55	7594.42		197		
	58–68	51		7625.87		199		
QRS (ms)	36–46	73	15.07	30.14	0.54	2		0.58
	47–57	76	27.93	5503.53		197		
	58–68	51		5533.68		199		
QT (ms)	36–46	73	229.65	459.29	1.11	2		0.33
	47–57	76	207.84	40,945.88		197		
	58–68	51		41,405.18		199		
Tp-e (ms)	36–46	73	44.34	88.69	0.59	2		0.55
	47–57	76	74.03	14,584.70		197		
	58–68	51		14,673.39		199		
Tp-e/QT	36–46	73	0.00	0.00	0.35	2		0.71
	47–57	76	0.00	0.038		197		
	58–68	51		0.038		199		
SS	36–46	73				2	4.76	0.093
	47–57	76						
	58–68	51						
SDS	36–46	73				2	1.54	0.462
	47–57	76						
	58–68	51						
ISES	36–46	73				2	0.76	0.681
	47–57	76						
	58–68	51						

Abbreviations: HR: Heart rate, SS: Spirituality Score, SDS: Stress Distress Score, ISES: Insufficient Self-Efficacy Score.

**Table 5:** Multivariate linear regression analysis of predictors of HRV and ECG parameters.

Variable Model 1: SDNN (R^2^ = 0.32)	Β	SE	*p*
Spirituality (SS)	0.23	0.05	<0.001
Stress (SDS)	−0.19	0.06	0.002
ISES	−0.12	0.05	0.041
Age	−0.08	0.04	0.12
BMI	−0.09	0.04	0.09
Hypertension	−0.28	0.06	<0.001
**Variable** **Model 2: RMSSD (R^2^ = 0.28)**	**Β**	**SE**	***p***
Spirituality (SS)	0.21	0.06	<0.001
Stress (SDS)	−0.18	0.06	0.003
ISES	−0.11	0.05	0.048
Age	−0.06	0.04	0.18
BMI	−0.07	0.04	0.14
Hypertension	−0.25	0.06	<0.001
**Variable** **Model 3: Tp-e (R^2^ = 0.35)**	**Β**	**SE**	***p***
Spirituality (SS)	−0.20	0.05	<0.001
Stress (SDS)	0.17	0.06	0.004
ISES	0.10	0.05	0.06
Age	0.09	0.04	0.11
BMI	0.08	0.04	0.13
Hypertension	0.30	0.06	<0.001
**Variable** **Model 4: Tp-e/QT (R^2^ = 0.31)**	**Β**	**SE**	***p***
Spirituality (SS)	−0.18	0.05	0.002
Stress (SDS)	0.16	0.06	0.006
ISES	0.11	0.05	0.049
Age	0.07	0.04	0.15
BMI	0.08	0.04	0.12
Hypertension	0.27	0.06	<0.001

Abbreviations: SS: Spirituality Score; SDS: Stress Distress Score; ISES: Insufficient Self-Efficacy Score.

## Data Availability

The datasets generated and analyzed during the current study are available from the corresponding author on reasonable request.
